# Longer preserved urethral length in robot‐assisted radical prostatectomy significantly contributes to post‐operative urinary continence recovery

**DOI:** 10.1002/bco2.128

**Published:** 2021-11-12

**Authors:** Satoshi Ando, Jun Kamei, Masahiro Yamazaki, Toru Sugihara, Tomohiro Kameda, Akira Fujisaki, Shinsuke Kurokawa, Tatsuya Takayama, Tetsuya Fujimura

**Affiliations:** ^1^ Department of Urology Jichi Medical University Shimotsuke Japan

**Keywords:** prostate cancer, robot‐assisted radical prostatectomy, urinary continence

## Abstract

**Objectives:**

To assess the relationship between the surgical procedure of robot‐assisted radical prostatectomy (RARP) and urinary continence recovery by reviewing the video database.

**Methods:**

Video and data about men diagnosed with prostate cancer and underwent RARP were extracted and reviewed. Preserved urethral length (PUL) was semi‐quantitatively measured using the lateral width of a 16‐Fr urethral balloon catheter while cutting the urethra on a video screen. In addition, by reviewing intraoperative RARP video database, other surgical skill outcomes were also collected. Kaplan–Meier analysis with log‐rank test was used to compare the urinary continence recovery rate, stratified by the PUL. Univariate and multivariate analyses were performed using the Cox proportional hazards model, and *p*‐values of <0.05 were considered significant.

**Results:**

The number of patients included in this study was 213. In univariate analysis, a PUL of ≥16 mm, a body mass index of <23.1 kg/m^2^ and a resected prostate volume of <44.3 g were statistically significant factors that influenced urinary continence recovery [hazard ratio (HR) 1.58, *p* = 0.036; HR 0.67, *p* = 0.021; and HR 0.58, *p* = 0.005, respectively]. Those factors also remained statistically significant in the multivariate analysis (HR 1.87, *p* = 0.022; HR 0.54, *p* = 0.001; and HR 0.57, *p* = 0.005, respectively). One year post‐operatively, the recovery rate from urinary continence was 79.0% for patients with a PUL of ≥16 mm and 66.5% for patients with a PUL of <16 mm.

**Conclusion:**

These results suggest that patients with longer PUL in RARP have a significantly higher rate of post‐operative urinary continence recovery.

## 1 INTRODUCTION

Prostate cancer is a malignant tumour that most commonly occurs in men. In Japan, 91 215 men developed prostate cancer in 2017. Among all carcinomas, the number of patients with prostate cancer was the highest.^1^ In 2018, 12 250 men died of prostate cancer, making it the sixth among all cancer deaths.^2^

Surgery or radiation therapy is considered for the treatment of localized prostate cancer. In Japan, robot‐assisted radical prostatectomy (RARP) has been performed in the health insurance since 2012, and the number of cases is increasing. Compared with conventional open surgery, RARP allows delicate surgical procedures with non‐shaky forceps in an enlarged field of view. Despite improvements in such surgical techniques, incontinence occurring after RARP remains a significant, quality‐of‐life complication.^3^

Previous studies have examined factors associated with achieving urinary continence after a radical prostatectomy, including patient selection; preoperative pelvic floor muscle exercises; preoperative membranous urethral length (MUL); operative procedures such as intraoperative preservation, reconstruction and reinforcement; postoperative pelvic floor muscle exercises; and duration of urinary catheter placement.^4^ Preoperative MUL was measured by magnetic resonance imaging (MRI) of the prostate. However, it is not always possible to preserve the original membranous urethra in a patient with a long MUL. For achieving urinary continence, it was conceived that it was important to retain the length of the membranous urethra by surgery, and not by preoperative MUL. Therefore, this study aimed to clarify factors associated with urinary continence recovery using clinical records and RARP video databases, especially PUL, which was measured semi‐quantitatively by reviewing RARP video. It is hoped that the analysis results will lead to an improved urinary continence recovery rate.

## 2 PATIENTS AND METHODS

### 2.1 Patients

This retrospective study was approved by the institutional review board (IRB) of our institute (IRB number: A19‐199). Data of patients diagnosed with prostate cancer and underwent RARP at our institution between March 2016 and October 2019 were extracted.

Baseline patient characteristics and perioperative and pathological outcomes were obtained from medical records, including age, body mass index (BMI), preoperative prostate‐specific antigen (PSA), day of the surgery, presence or absence of urinary continence after RARP, number of days to achieve urinary continence, date of last consultation, total operative time, console time, loss of blood volume, weight of the resected prostate, pathological T stage, surgical margin status and follow‐up periods according to a previous literature.^5^ The day of continence recovery was registered when social continence was attained (a small 20‐ml pad required daily) according to previous literature.^5^ Urethral balloon catheters were removed in all patients on post‐operative Day 6. All patients were instructed to perform pelvic floor exercises post‐operatively.

**FIGURE 1:**
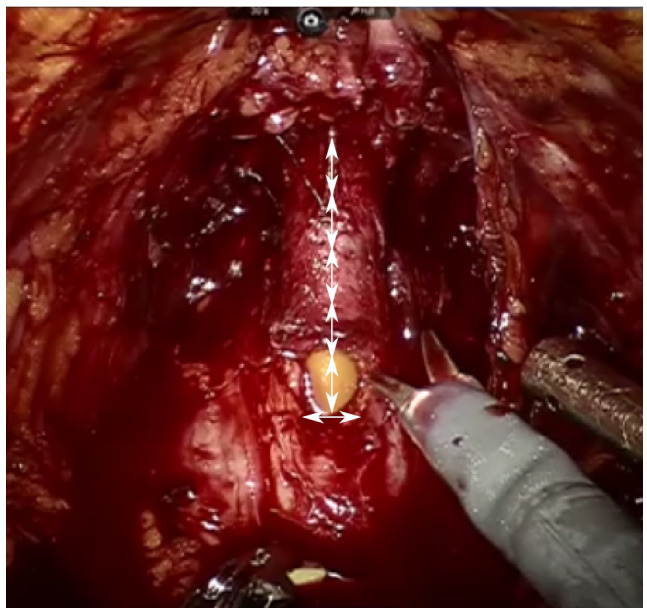
Using the width of the urethral catheter (16/3 mm) while making an incision in the urethra, the preserved membranous urethral length (PUL) was semi‐quantitatively measured on a video screen in increments of 0.5 lines. One arrow indicates the width of the 16‐Fr urethral balloon catheter. The PUL was determined to be 5 (approximately 26.7 mm)

By reviewing the intraoperative RARP video database, surgical skill outcomes such as preservation of cavernous nerves of the penis, puboprostatic ligament, endopelvic fascia and PUL; bunching suture of the dorsal vein complex (DVC); and vesicourethral anastomotic failure were also collected. Anastomotic failure was determined to be positive if there was a leakage in the leak test after anastomosis. PUL was measured as follows. A 16‐Fr urethral balloon catheter was placed in all patients preoperatively. Using the width of the urethral catheter (16/3 mm) while making an incision in the urethra, the remaining PUL was semi‐quantitatively measured on a video screen in increments of 0.5 lines. The measurement was performed under the condition that the prostate was pulled to the head side by the third arm. (Figure 1) For each patient's surgical video, two researchers independently evaluated the procedure. A total of five researchers (SA, TS, JK, TK and MY) who could perform RARP independently and were involved in the research. All researchers are certified specialist of the Japanese Urological Association. If the researchers had different views, a decision was made by a conference between the two researchers.

### 2.2 Surgical techniques of RARP

RARP was performed using a transperitoneal approach with six ports according to standard techniques, as previously described.^5^ In case of unilateral nerve sparing, the sparing side was determined from imaging findings of MRI and the positive site of prostate biopsy. Bilateral nerve sparing was performed when the positive site of prostate biopsy was only in the transition zone. We performed posterior reconstruction only. A total of nine surgeons performed RARP in the present study. One of them also participated in all operations as an instructive assistant.

### 2.3 Statistical analyses

The Wilcoxon rank sum test was used for continuous data, and the chi‐squared test was used for categorical data. Continence recovery rate curves were plotted using the Kaplan–Meier method and compared using the log‐rank test. Univariate and multivariate Cox proportional hazards models were used to analyse the correlation between clinical parameters and the continence recovery. The cut‐off values for age, BMI and resected prostatic volume were determined using receiver operating characteristic (ROC) curves (Figure S1). The cut‐off value for PUL was determined to be 16 mm by exploratory research from the results obtained. All statistical analyses were performed using the JMP Pro Version 14.0 software (SAS Institute Inc., Cary, NC, USA), with a *p*‐value of 0.05 considered to represent a significant difference.

## 3 RESULTS

Patient characteristics and perioperative findings are summarized in Table 1. The median age of patients, preoperative PSA concentration, BMI and the weight of resected prostate were 68.0 years, 7.03 ng/ml, 24.0 kg/m^2^ and 34.0 g, respectively. The median operative and console times of 228 and 176 min, respectively. The median amount of bleeding is 100 ml. With regard to nerve sparing, 23, 45 and 145 patients had bilateral, unilateral and non‐nerve‐sparing operations, respectively. The bunching of DVC was performed in 100 patients. With regard to endopelvic fascia, 64, 33 and 116 patients had bilateral, unilateral and non‐fascia‐sparing operation, respectively. With regard to puboprostatic ligaments, 27, 21 and 164 patients had bilateral, unilateral and non‐ligament‐sparing operations, respectively. Vesicourethral anastomotic failure occurred in 29 patients intraoperatively. The median PUL was 10.7 mm (two times the 16‐Fr balloon catheter width). The median post‐operative follow‐up period was 186 days (interquartile range: 7–349 days). The oncological data and post‐operative recurrence are summarized in Table 2.

**TABLE 1:** Patient characteristics and perioperative parameters

	All patients (*n* = 213)	Patients with PUL < 16 mm (*n* = 175)	Patients with PUL ≥ 16 mm (*n* = 38)	*p*‐value
Age, years, median (IQR)	68 (64–71)	67 (64–71)	68 (63.3–70.8)	
Body mass index, median (IQR)	24.0 (22.0–26.0)	24.0 (22.0–25.9)	23.9 (22.6–26.2)	0.63
Preoperative PSA, ng/ml, median (IQR)	7.03 (5.5–10.0)	7.06 (5.53–26.0)	7.02 (5.39–9.10)	0.2
Post‐operative prostate weight, g, median (IQR)	34.0 (28.2–43.2)	36.4 (28.3–43.3)	32.6 (27.5–43.0)	0.67
Operative time, minute, median (IQR)	228 (194–268)	229 (199–273)	214 (104–191)	0.78
Console time, minute, median (IQR)	176 (143–211)	184 (149–214)	159 (105–214)	0.0028^*^
Bleeding loss, ml, median (IQR)	100 (50–250)	100 (50–250)	100 (50–308)	0.0043^*^
Cavernous nerve sparing (%)				0.37
Bilateral	23 (10.8)	19 (10.9)	4 (10.5)	<0.0001^*^
Unilateral	45 (21.1)	24 (13.7)	21 (55.3)	
No	145 (68.1)	132 (75.4)	13 (34.2)	
DVC bunching (%)				
Yes	100 (46.9)	99 (56.6)	1 (2.6)	<0.0001^*^
No	113 (53.1)	76 (43.4)	37 (97.4)	
Endopelvic fascia sparing (%)				
Bilateral	64 (30.0)	43 (24.6)	21 (55.3)	<0.0001^*^
Unilateral	33 (15.5)	21 (12)	12 (31.6)	
No	116 (54.5)	111 (63.4)	5 (13.2)	
Puboprostatic ligament sparing (%)				
Bilateral	27 (12.7)	25 (14.3)	19 (50)	<0.0001^*^
Unilateral	21 (9.9)	8 (4.6)	7 (18.4)	
No	164 (77.0)	142 (81.1)	12 (31.6)	
Anastomotic failure (%)				
Yes	29 (13.6)	20 (11.4)	9 (23.7)	0.794
No	184 (86.4)	155 (88.6)	29 (76.3)	
PUL, mm, median (IQR)	10.7 (5.3–10.7)	8 (5.3–10.7)	16 (16–16)	

Abbreviations: DVC, dorsal vein complex; IQR, interquartile range; PSA, prostate‐specific antigen; PUL, preserved urethral length.

* Statistically significant.

**TABLE 2:** Pathological findings and post‐operative recurrence

	All patients (*n* = 213)	Patients with PUL < 16 mm (*n* = 175)	Patients with PUL ≥ 16 mm (*n* = 38)
Biopsy ISUP grade			
Grade 1, *n* (%)	22 (10.3)	19 (10.9)	3 (5.9)
Grade 2, *n* (%)	78 (36.6)	65 (37.1)	26 (51.0)
Grade 3, *n* (%)	52 (24.4)	46 (26.3)	6 (11.8)
Grade 4, *n* (%)	40 (18.8)	29 (16.6)	11 (21.6)
Grade 5, *n* (%)	21 (9.9)	16 (9.1)	5 (9.8)
Pathological T stage, *n* (%)			
pT2a	40 (18.8)	30 (17.1)	10 (26.3)
pT2b	2 (0.9)	2 (1.1)	0 (0)
pT2c	122 (57.3)	101 (57.7)	21 (55.3)
pT3a	36 (16.9)	31 (17.7)	5 (13.2)
pT3b	11 (5.2)	10 (5.7)	1 (2.6)
pT4	2 (0.9)	1 (0.6)	1 (2.6)
Positive surgical margins, *n* (%)			
All stages, *n* (%)	71 (33.3)	61 (34.9)	9 (23.7)
pT2, *n* (%)	41 (25.0)	35 (26.3)	6 (19.4)
pT3, *n* (%)	28 (60.0)	26 (63.4)	2 (33.3)
Biochemical recurrence, *n* (%)	11 (5.2)	7 (4.0)	4 (10.5)
Therapy after biochemical recurrence			
Radiation therapy, *n* (%)	4 (36.4)	1 (14.3)	3 (75.0)
Hormonal therapy, *n* (%)	4 (36.4)	3 (42.9)	1 (25.0)
Radiation + hormone therapy, *n* (%)	3 (27.3)	3 (42.9)	0 (0)

**FIGURE 2:**
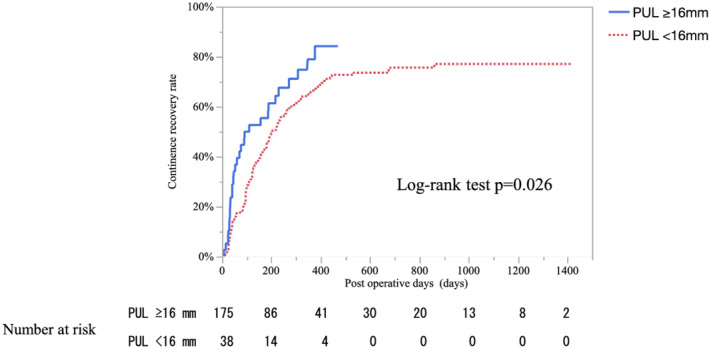
Continence recovery rate curves were plotted using the Kaplan–Meier method. A preserved urethral length ≥16 mm was a statistically significant factor that influenced urinary continence recovery (*p* = 0.026)

To determine the cut‐off value for PUL, a bar graph was made firstly to show continence recovery rate by PUL shown in Figure S2. Therefore, it was considered that there might be a difference in urinary continence when the PUL was around 16 mm, and when the Kaplan–Meier curve was drawn for the cumulative rates of urinary continence with respect to the PUL in an exploratory manner, a significant difference was seen at the border of 16 mm. Then, we decided to apply 16 mm as the cut‐off value. A PUL of ≥16 mm was a statistically significant factor that influenced urinary continence recovery (log‐rank test, *p* = 0.026) (Figure 2). The cumulative continence recovery rates at 12 months were 79.0% for patients with ≥16‐mm PUL and 66.5% for patients with <16‐mm PUL (Figure 2). The Kaplan–Meier curve is shown to show urinary continence recovery rate by nerve sparing methods (Figure S3).

Univariate analysis of Cox proportional hazards model demonstrated that a PUL of ≥16 mm, a BMI of <23.1 kg/m^2^ and a resected prostatic volume of <44.3 g were significantly associated for the recovery of continence. The hazard ratio for a PUL of ≥16 to <16 mm was 1.58 [95% confidence interval (CI), 1.03–2.34; *p* = 0.036], the hazard ratio for a BMI of <23.1 to ≥23.1 kg/m^2^ was 0.75 (95% CI, 0.50–1.18; *p* = 0.021), and the hazard ratio for a resected prostatic volume of <44.3 to ≥44.3 g was 0.58 (95% CI, 0.38–2.34; *p* = 0.005). Multivariate analysis of Cox proportional hazards model also demonstrated that a PUL of ≥16 mm, a BMI of <23.1 kg/m^2^ and a resected prostatic volume of <44.3 g were significantly associated for the recovery of continence. The hazard ratio for a PUL of ≥16 to <16 mm was 1.87 (95% CI, 1.10–3.12; *p* = 0.022), the hazard ratio for a BMI of <23.1 to ≥23.1 kg/m^2^ was 0.54 (95% CI, 0.38–0.78; *p* = 0.001), and the hazard ratio for a resected prostatic volume of <44.3 to ≥44.3 g was 0.57 (95% CI, 0.38–0.85; *p* = 0.005) (Table 3).

**TABLE 3:** Cox proportional hazards model

	Univariate			Multivariate		
	HR	95% CI	P value	HR	95% CI	*p*‐value
Age (≥62 vs. <62)	0.75	0.50–1.18	0.204	0.71	0.46–1.13	0.14
BMI (≥23.1 vs. <23.1)	0.67	0.49–0.94	0.021*	0.54	0.38‐0.78	0.001^*^
Preservation of cavernous nerves of penis (yes vs. no)	1.11	0.78–1.56	0.596	0.63	0.38–1.04	0.073
Preservation of endopelvic fascia (yes vs. no)	1.12	0.81–1.55	0.492	1.29	0.71–2.37	0.405
DVC bunching (yes vs. no)	0.99	0.71–1.37	0.956	0.85	0.49–1.49	0.578
Preservation of puboprostatic ligament (yes vs. no)	1.32	0.88–1.93	0.171	1.03	0.62–1.71	0.897
Anastomosis failure (yes vs. no)	0.75	0.49–1.19	0.213	0.63	0.39–1.06	0.081
PUL (≥16 vs. <16 mm)	1.58	1.03–2.34	0.036*	1.87	1.10‐3.12	0.022^*^
Resected prostatic volume (≥44.3 vs. <44.3 g)	0.58	0.38–2.34	0.005*	0.57	0.38‐0.85	0.005^*^

Abbreviations: BMI, body mass index; DVC, dorsal vein complex; PUL, preserved urethral length.

* Statistically significant.

## 4 DISCUSSION

In this study, we examined the surgical technique of RARP using the video database and analysed the relationship between the surgical technique and urinary continence recovery using univariate and multivariate analyses, which demonstrated that a PUL of ≥16 mm, a BMI of <23.1 kg/m^2^ and a resected prostatic volume of <44.3 g were significant for urinary continence recovery. Although BMI and prostatic volume are already determined preoperatively, the possibility of longer preservation of the membranous urethra depends on the surgical techniques.

A previous study had changed the surgical technique of the prostatic apex to maximize preservation of the membranous urethra. As a result, the urinary continence recovery rate at an early stage (30‐day) and long term (1 year) is improved.^6^ Inconsistent with the results of the present study, the PUL was not investigated. Longer innate MUL on preoperative MRI has also been reportedly associated with early post‐operative recovery from urinary continence.^6^, ^7^, ^8^, ^9^ Significant recovery has been reported for an MUL of >14^6^ and >10.5 mm.^7^ The MUL attached to the resected specimen has been reported as a predictor of post‐operative incontinence.^10^ This study did not investigate the MUL and early recovery of urinary continence on preoperative MRI or resected specimens. The measurement method of MUL on MRI is different among researchers and thus cannot be compared; however, longer MUL is suggested to contribute to post‐operative urinary continence. However, it is not always possible to preserve the original membranous urethra in a patient with a long MUL. Although there has been a study investigating the relationship between longer MUL during surgery and post‐operative urinary continence,^6^ few studies have examined how long it was preserved.^11^ In this study, the measurement of urethral length was semi‐quantitative, but the relationship between PUL and urinary continence was similar to previous reports, and evidence for their relationship was accumulated. The long functional urethral length and high urethral closure pressure have been speculated as good reasons for continence.^12^ A previous study investigated the male and female urethral function using transrectal/transvaginal ultrasonography while micturiting.^13^ The proximal 4/5 length of the urethra was surrounded by a thick muscle unit, a unit developed only on the anterior and lateral sides of the urethra, and the posterior side was firmly fixed to the vaginal wall by very thin muscles. While maintaining continence, the whole muscle layer swelled sufficiently to close the urethral lumen evenly. The initial motion was not a bladder contraction, but an active urethral lumen opening by reducing the muscle thickness. In men, muscles similar to those found around the female urethra are present in the prostatic urethra, and similar muscle movements were seen during urination. The distal end of the muscle was thought to be connected to a muscle known as the external urethral sphincter muscle at the apex of the prostate. When cutting the urethra at the apex of the prostate, preserving this muscle as much as possible while preventing positive margins in prostate cancer may be important, when considering urinary continence recovery.

In a meta‐analysis of postoperative incontinence after a cavernous nerve‐sparing radical prostatectomy, bilateral nerve sparing was associated with lower incontinence rate than unilateral nerve sparing; however, the rate was significantly lower only 1 year post‐operatively.^14^ The lack of thermal injury and greater tissue preservation around the urethra and pelvic floor during nerve sparing may explain the association.^14^ Although cavernous nerve preservation has been suggested to contribute to the long‐term rather than early urinary continence recovery, the results of both univariate and multivariate analyses of the relationship between cavernous nerve preservation and urinary continence recovery were not significant in this study. Because only a few patients had preserved cavernous nerve of the penis, the possibility of the absence of the statistical significance and the possibility that the potency was low preoperatively were considered.

Although no randomized controlled trials (RCTs) have compared different surgical approaches in RARP,^15^ subgroup analyses of RCTs have shown that bladder neck preservation leads to continence recovery in open surgery and RARP.^16^ Bladder neck preservation has been reported to contribute to urinary continence in the early post‐operative period (3–6 months); however, no significant difference was observed in the final urinary continence, and no advantage was observed with respect to puboprostatic ligament preservation.^17^ In contrast, when patients were divided into two groups according to whether the puboprostatic ligament was preserved or not during a nerve‐sparing endoscopic extraperitoneal radical prostatectomy, the urinary continence recovery rate has been reportedly significantly higher in the puboprostatic ligament preservation group.^18^ In the PUL ≥ 16 mm group, there were significantly more cases with preservation of puboprostatic ligands. In another study to examine the effect of reconstruction of the puboprostatic ligaments on the social continence, it was found that there was not a significant difference in the preservation of the puboprostatic ligaments group or not.^19^ In this study, the presence or absence of puboprostatic ligament preservation was not associated with urinary continence. There was a possibility that the preservation of the puboprostatic ligaments did not affect the overall continence. In addition, this study did not consider bladder neck preservation, and thus, we would like to modify the surgical method in future studies.

The preservation of the endopelvic fascia has been shown to help early urinary continence recovery.^20^, ^21^, ^22^ The lack of thermal injury and greater tissue preservation around the urethra and pelvic floor while nerve sparing may explain the association.^14^ In this study, the presence or absence of endopelvic fascia preservation was not associated with urinary continence recovery.

Cutting DVC after DVC bunching to control bleeding from DVC may injure the surrounding levator ani muscles. In the pneumoperitoneum, even if DVC is cut without ligation, the amount of bleeding is reduced, and selective suture ligation is possible.^23^ In a report on RARP in which DVC was cut with scissors and the cut end was sutured selectively, urinary continence significantly improved at 5 months post‐operatively; however, no significant difference was observed at 12 months post‐operatively.^24^ Selective suture ligation may contribute to the early urinary continence recovery post‐operatively. In this study, DVC bunching was performed at first; however, the operative method was changed in the middle, and DVC was cut without bunching, making selective suture ligation. However, no significant difference was observed in the urinary continence recovery using the DVC treatment method in univariate and multivariate analyses.

The present study has several limitations. First, although the design was retrospective, it was conducted at a single institution. External validation using another data set is needed in the future. Second, PUL was measured semi‐quantitatively. PUL should be precisely measured in future studies. Third, measurement of PUL may result in a measurement error due to the amount of force of cephalad retraction of the prostate with the third robotic arm. Lastly, in the population sample, the small prostate and very high rate of non‐nerve‐sparing RARP are of concern when considering the rate of urinary continence.

## 5 CONCLUSION

These results suggest that patients with longer PUL in RARP have a significantly higher post‐operative urinary continence recovery rate. The development of the surgical procedure that PUL longer seems to have great impact on the urinary continence.

## CONFLICT OF INTEREST

None declared.

## AUTHOR CONTRIBUTIONS

All authors on the manuscript were involved with operating patients. Satoshi Ando, Jun Kamei, Masahiro Yamazaki, Toru Sugihara and Tomohiro Kameda were involved with data collection. Satoshi Ando and Tetsuya Fujimura led the manuscript effort.

## Supporting information


**Figure S1.** ROC curve

**Figure S2.** Continence recovery rate by preserved urethral length

**Figure S3.** Continence recovery rate by nerve‐sparing methods
