# Supplementary figures and images for: Longer preserved urethral length in robot‐assisted radical prostatectomy significantly contributes to post‐operative urinary continence recovery
Source: BJUI Compass. 2021 Nov 12;3(2):184–90. doi: 10.1002/bco2.128 (PMC8988697; doi:10.1002/bco2.128)

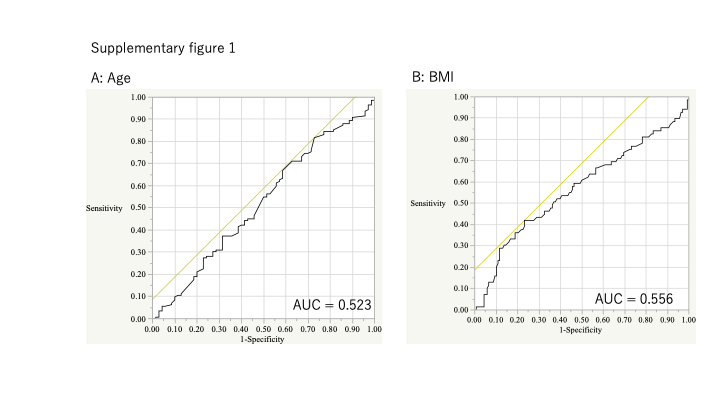

Supplement: Supplementary file 1 — Figure S1. ROC curve [file BCO2-3-184-s003.png]

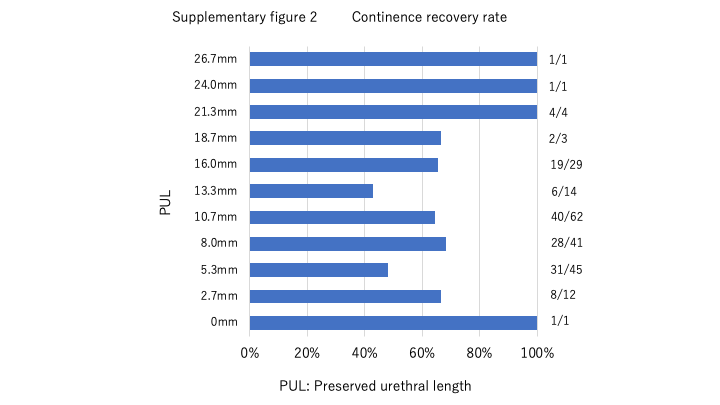

Supplement: Supplementary file 2 — Figure S2. Continence recovery rate by preserved urethral length [file BCO2-3-184-s001.png]

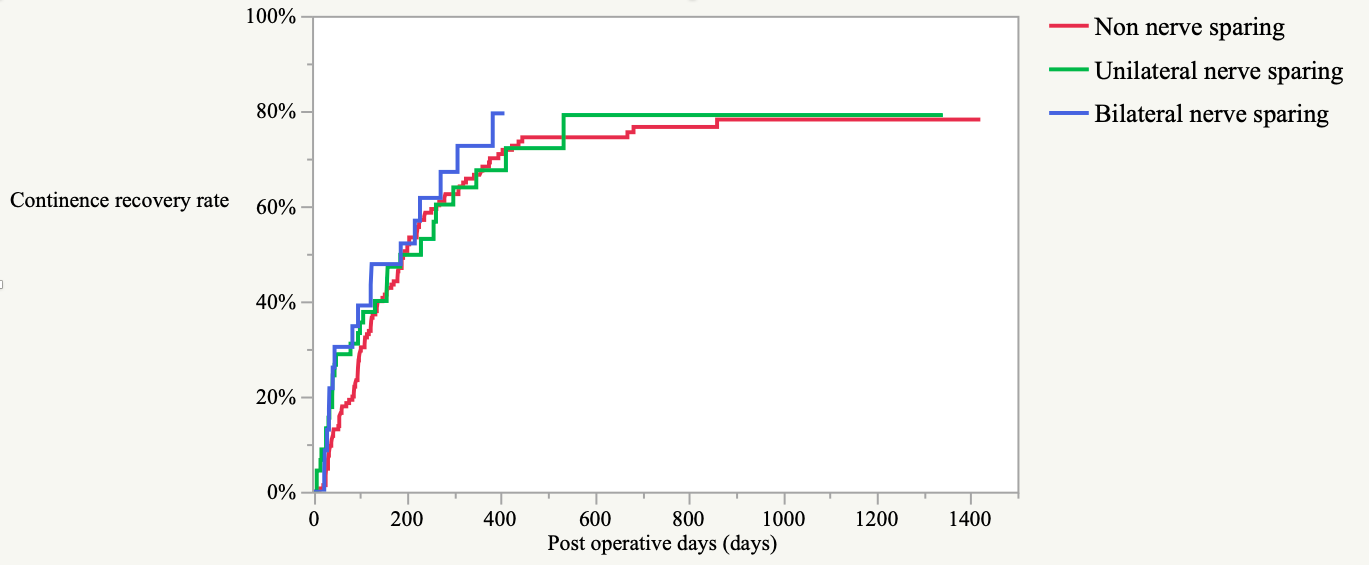

Supplement: Supplementary file 3 — Figure S3. Continence recovery rate by nerve‐sparing methods [file BCO2-3-184-s002.png]
